# The Relationship of ABO and Rh Blood Group Types With Severe COVID-19 Disease Mortality in ICU Patients: Insights From a Single-Center Experience in Southern Saudi Arabia

**DOI:** 10.7759/cureus.50935

**Published:** 2023-12-22

**Authors:** Ali Al Bshabshe, Mushary Alqahtani, Khaled A Amer, Najla A Al-jahash, Abduallh S Thwab, Faleh S Alshahrani, Abdullah Saad aldarani alshahrani, Osama A Asiri, Faisal N Abughazalah, Ahmed Ali A Khuzayyim, Faisal Altumaihi, Turki khawaji, Ayman Algaide, Moyed Almontasheri

**Affiliations:** 1 Critical Care, King Khalid University, Abha, SAU; 2 Internal Medicine, Armed Forces Hospitals Southern Region, Khamis Mushait, SAU; 3 Medicine and Surgery, King Khalid University, Abha, SAU; 4 Internal Medicine, Asir Central Hospital, Abha, SAU; 5 Internal Medicine, King Abdullah Medical City, Makkah, SAU; 6 General Practice, Primary Healthcare Center, South Khaybar, Abha, SAU; 7 General Practice, King Abdullah Hospital, Bishah, SAU; 8 Medical School, King Khalid University, Abha, SAU; 9 Intensive Care Unit, King Fahad General Hospital, Jeddah, SAU

**Keywords:** mechanical ventilator, ards, critical care, blood group, covid-19

## Abstract

Introduction

The global COVID-19 pandemic has triggered an unprecedented public health crisis, emphasizing the need to understand factors influencing disease outcomes. This study explores the role of genetic variations in blood group antigens, particularly ABO and RhD, in shaping mortality rates among critically ill COVID-19 patients in the southern region of Saudi Arabia.

Methods

Utilizing a retrospective, noninterventional approach, we analyzed medical records of 594 COVID-19 patients admitted to the intensive care unit (ICU) at Aseer Central Hospital from August 2020 to April 2021. The cohort, with a mean age of 60.5 years, consisted of a predominantly male population.

Results

The study encompassed a diverse age range of 18 to 103 years, with a mean age of 60.5 ± 17.3 years. Of the 594 patients, 398 (67%) were male, and only 5 (0.8%) had a history of smoking. Blood group distribution revealed 275 (48.4%) with O-, 189 (33.3%) with A+, and 51 (9%) with AB- types. Predominant chronic conditions included diabetes mellitus (35.5%). Tragically, 320 patients (54.6%) experienced mortality, with a 100% mortality rate for the B+ blood group and 92.9% for O- blood group.

Conclusion

This analysis establishes significant statistical links, underscoring the pivotal role of blood type, particularly the Rh factor, in influencing mortality risk among critically ill COVID-19 patients. These findings contribute valuable insights into risk stratification and personalized care for severe cases, emphasizing the importance of genetic considerations in understanding disease outcomes.

## Introduction

The global outbreak of coronavirus disease 2019 (COVID-19), caused by the severe acute respiratory syndrome coronavirus 2 (SARS-CoV-2) led to an unprecedented public health crisis. This rapidly spreading virus posed significant challenges to healthcare systems across the globe, especially in managing critically ill patients requiring admission to intensive care units (ICUs) [1,2]. Understanding the factors that contribute to disease severity and mortality among these severely affected COVID-19 patients is of utmost importance for refining patient care strategies and effectively allocating resources.

Among the myriad factors that influence disease outcomes, genetic and immunological considerations have come to the fore [3]. Genetic variations in blood group antigens, notably ABO and RhD (Rh factor), have been implicated in influencing susceptibility to infectious diseases and modulating responses to viral infections. These antigens, encoded by the ABO and Rh genes, exhibit striking population-specific distributions [4-6].

Extensive literature insights highlight the multifaceted involvement of blood group antigens in various physiological mechanisms, encompassing immune response modulation, coagulation dynamics, and viral attachment. These revelations have sparked the hypothesis that blood group antigens could potentially impact the trajectory of COVID-19 [7]. Furthermore, mounting evidence underscores the potential influence of the ABO blood group on both susceptibility and severity of SARS-CoV-2 infection. Additionally, the presence of Rh antigens on red blood cells adds another layer to the intricate interaction among immunity, inflammation, and the progression of the disease [8,9].

While studies in the Middle East (and Saudi Arabia in particular) have hinted at potential links between blood group antigens and COVID-19 severity [10,11], the specific association between blood group, Rh type, and mortality in ICU-admitted COVID-19 patients from the southern region remains underexplored. The primary objective of this study is to elucidate whether specific blood group antigens and Rh types are associated with differential mortality rates among critically ill COVID-19 patients admitted to the ICU in the southern region of Saudi Arabia.

## Materials and methods

Our study investigated COVID-19 ICU admissions at Aseer Central Hospital in the southern region of Saudi Arabia. Using a retrospective, noninterventional approach, we analyzed medical records of COVID-19 patients admitted to the ICU, particularly those requiring ventilation. Our cohort included patients aged 18 and above with SARS-CoV-2 infection confirmed through real-time polymerase chain reaction (RT-PCR) tests. Data collection spanned from August 2020 to April 2021, resulting in a final sample of 594 patients after thorough data cleansing and exclusions. We explored variables such as demographics, comorbidities, and ICU interventions, with a primary focus on patient mortality and its potential association with blood group types. This study contributes valuable insights into COVID-19's impact in the ICU context, shedding light on potential connections between blood group types and patient outcomes. Ethical approval was secured from the Institutional Review Board at King Khaled University (approval number ECM#2023-3103).

Following data extraction, a comprehensive process of revision, coding, and input into the IBM SPSS version 26 software (IBM Corp., Armonk, NY) was conducted. Statistical analyses employed two-tailed tests, with significance set at a P-value less than 0.05. Descriptive statistics, including mean and standard deviation, were employed for scale variables and continuous measures such as laboratory findings. Meanwhile, categorical variables, encompassing personal data and blood group, were analyzed using frequency and percentage. Graphical representation was used for comorbidity and mortality rate exploration. In order to explore mortality factors and associations, cross-tabulation was performed, with the chi-square test and exact test employed to compare categorical variables, particularly for smaller sample sizes. Furthermore, logistic regression analysis was applied to ascertain the extent of the relationship between blood group, Rh type, and patients' mortality rate.

## Results

A comprehensive cohort of 594 COVID-19 patients, who were admitted to the ICU at Aseer Central Hospital (ACH), formed the foundation of our study. This diverse group ranged in age from 18 to 103 years, with a mean age of 60.5 ± 17.3 years. Among them, 398 individuals (67%) were male, and a mere 5 (0.8%) had a history of smoking. Remarkably, over half of these patients displayed positive blood cultures. Concerning blood groups, the distribution revealed 275 (48.4%) with O-, 189 (33.3%) with A+, and 51 (9%) with AB- blood types (Table 1).

**Table 1:** Personal characteristics of covid patients admitted to Intensive Care Unit, Aseer Central Hospital, Saudi Arabia

Personal data	n	%
Age in years		
< 40	72	12.1%
40-59	193	32.5%
60-69	127	21.4%
70+	202	34.0%
Mean ± SD	60.5 ± 17.3	
Gender		
Male	398	67.0%
Female	196	33.0%
Smoking		
No	589	99.2%
Yes	5	.8%
Positive Blood Culture		
No	252	42.4%
Yes	334	56.2%
Blood group		
A+	24	4.2%
A-	189	33.3%
B+	2	.4%
B-	9	1.6%
AB+	3	.5%
AB-	51	9.0%
O+	15	2.6%
O-	275	48.4%

**Figure 1:**
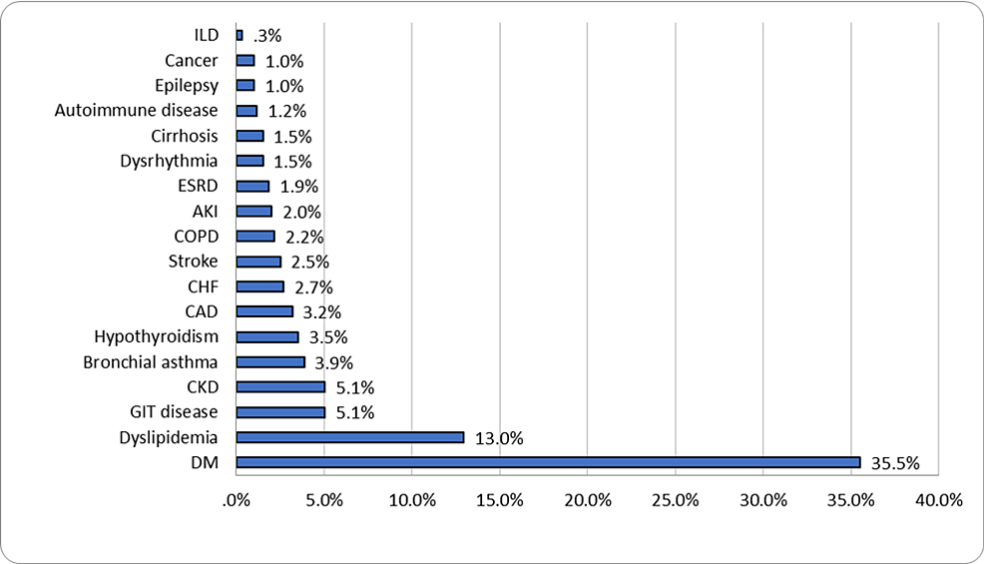
Chronic Diseases Among COVID Patients Admitted to Intensive Care Unit, Aseer Central Hospital, Saudi Arabia ILD: intestinal lung disease; ESRD: end-stage renal disease; AKI: acute kidney injury; COPD: chronic obstructive pulmonary disease; CHF: congestive heart failure; CAD: coronary artery disease; CKD: chronic kidney disease; GIT: gastrointestinal; DM: diabetes mellitus

Figure 1 succinctly depicts the prevalence of chronic diseases within this patient cohort. The most frequently observed chronic health conditions included diabetes mellitus in 211 patients (35.5%), dyslipidemia in 77 patients (13%), gastrointestinal disorders in 30 patients (5.1%), chronic kidney disease in 30 patients (5.1%), bronchial asthma in 23 patients (3.9%), and hypothyroidism in 21 patients (3.5%). Table 2 presents the collective laboratory findings of COVID-19 patients admitted to the ICU at Aseer Central Hospital (ACH). The table displays notable metrics, including an average blood glucose level (BGL) of 276.7 ± 129.1 mg/dl, creatinine at 2.6 ± 1.9 g/dl, erythrocyte sedimentation rate (ESR) at 62.8 ± 35.6, and C-reactive protein (CRP) at 60.4 ± 62.9.

**Table 2:** Laboratory Findings Among COVID Patients Admitted to the Intensive Care Unit, Aseer Central Hospital, Saudi Arabia Hb: hemoglobin, WBCs: white blood cells, LDL: low-density lipoprotein; HDL: high-density lipoprotein, ESR: erythrocyte sedimentation rate; CRP: C-reactive protein, BGL: blood glucose level

Laboratory findings	Mean	SD
Creatinine	2.6	1.9
Hb	13.3	2.9
WBCs	19.9	12.0
Platelets	131.8	132.1
LDL	78.3	59.4
HDL	35.1	17.4
ESR	62.8	35.6
CRP	60.4	62.9
BGL	276.7	129.1
Total bilirubin	1.8	2.1

**Figure 2:**
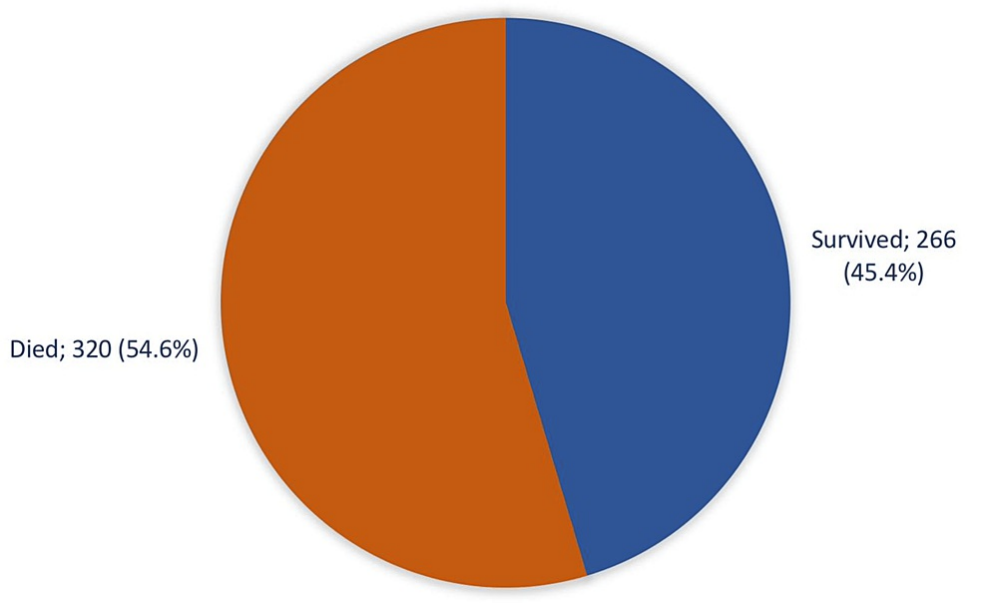
Death Rate Among COVID Patients Admitted to the Intensive Care Unit, Aseer Central Hospital, Saudi Arabia

Figure 2 graphically illustrates the mortality outcomes among the ICU-admitted COVID-19 patients. From the study's cohort, 320 patients (54.6%) unfortunately experienced mortality, while 266 patients (45.4%) emerged as survivors.

In this study, a significant correlation was found between age and mortality, with 150 patients (75%) aged 70 years or more succumbing to the disease compared to only 16 patients (22.5%) of those under 40 years (P=.001). Patients with positive culture results also had a higher mortality rate of 225 patients (67.4%) compared to 95 patients (37.7%) for those with negative culture findings (P=.001). Other factors examined did not show significant relations with COVID-19 patients' mortality (Table 3). Exploring the link between COVID-19 patients' blood group and mortality, a compelling pattern emerged. The mortality rate for patients with blood group B+ (2 patients) was 100%, while those with O- blood group (143 patients) exhibited a mortality rate of 92.9%. Similarly, A+ and A- blood groups experienced mortality rates of 62.5% and 58.5% (15,110 patients), respectively. This analysis demonstrated significant statistical associations (P=.016), underscoring the potential influence of blood type on mortality risk. Notably, only the Rh factor displayed a significant connection with mortality, as Rh-positive patients showed an almost twofold higher likelihood of mortality compared to Rh-negative patients (OR=2.0; 95% CI: 1.1-3.8; P=.046) (Tables 4-5).

**Table 3:** Factors Associated With Mortality Among COVID Patients Admitted to the Intensive Care Unit, Aseer Central Hospital, Saudi Arabia P: Pearson x^2^ test; ^$^ Exact probability test; ^*^P < 0.05 (significant)

Factors	Death				p-value
	No		Yes		
	n	%	n	%	
Age in years					.001*
< 40	55	77.5%	16	22.5%	
40-59	110	58.2%	79	41.8%	
60-69	51	40.5%	75	59.5%	
70+	50	25.0%	150	75.0%	
Gender					.221
Male	171	43.6%	221	56.4%	
Female	95	49.0%	99	51.0%	
Smoking					.510^$^
No	263	45.3%	318	54.7%	
Yes	3	60.0%	2	40.0%	
Diabetes mellitus					.578
No	167	44.5%	208	55.5%	
Yes	99	46.9%	112	53.1%	
Culture					.001*
No	157	62.3%	95	37.7%	
Yes	109	32.6%	225	67.4%	

**Table 4:** Relation Between COVID Patients’ Blood Group and Mortality Among Patients Admitted to the Intensive Care Unit, Aseer Central Hospital, Saudi Arabia P: Pearson x^2^ test; ^*^P < 0.05 (significant)

Blood group	Death				p-value
	No		Yes		
	n	%	n	%	
A+	9	37.5%	15	62.5%	.016*
A-	78	41.5%	110	58.5%	
B+	0	0.0%	2	100.0%	
B-	4	50.0%	4	50.0%	
AB+	3	100.0%	0	0.0%	
AB-	27	54.0%	23	46.0%	
O+	1	7.1%	13	92.9%	
O-	129	47.4%	143	52.6%	

**Table 5:** Relation Between COVID Patients’ Blood Group and RH With Mortality Among Patients Admitted to the Intensive Care Unit, Aseer Central Hospital, Saudi Arabia P: Pearson x^2^ test; ^*^P < 0.05 (significant)

Blood group	Death				p-value	OR (95% CI)
	No		Yes			
	n	%	n	%		
A	87	41.00%	125	59.00%	0.227	1.8 (0.5-2.9)
B	4	40.00%	6	60.00%		1.9 (0.4-2.9)
AB	30	56.60%	23	43.40%		1
O	130	45.50%	156	54.50%		1.7 (0.6-3.1)
Rh					.046*	
Positive	13	30.20%	30	69.80%		2.0 (1.1-3.8)*
Negative	238	45.90%	280	54.10%		

## Discussion

Researchers have found evidence suggesting that blood groups could potentially be a risk factor for severe COVID-19. In light of this, our study endeavors to offer valuable insights into the associations between blood group antigens, Rh types, and mortality rates in severely critical COVID-19 cases admitted to the ICU in ACH southern Saudi.

Our study mainly comprised males, consistent with national and global trends. Additionally, a significant portion of our ICU COVID-19 patients were aged 60 or older, mirroring findings from local and global studies [12-15]. Older age is linked to more severe disease and complications, driven by factors like comorbidities, weakened immunity, and increased vulnerability. Recognizing these risks is crucial for tailored care and better outcomes in this vulnerable group [12,16,17]. Also, the study revealed that over a third of patients had DM, consistent with the DM2 prevalence among Saudi individuals over 55. This aligns with our patient age group. Notably, our DM rate was higher than some regional studies in the eastern and western regions (20-30%) that didn't exclusively focus on ICU patients, but in line with another cohort study where almost half of COVID patients had DM [18,12,11]. This study unveiled a significant mortality rate among severely ill COVID-19 patients admitted to the intensive care unit (ICU) and requiring mechanical ventilation. An important correlation was observed between age and mortality in this group. Furthermore, patients who tested positive for the concomitant bacterial culture exhibited a heightened mortality rate. These findings are consistent with the results of various local studies conducted in Saudi Arabia, as well as a comprehensive analysis encompassing multiple countries [14-16,19,20].

Erythrocytes carry ABO blood group antigens (A, B, H) and also determine the Rh type. ABO blood groups categorize into four basic types: A, B, AB, and O. Recent research suggests a potential link between ABO blood types and COVID-19, possibly affecting susceptibility, severity, and disease behavior in infected individuals [21,22]. Close to 50% of the patients possess blood type O, which stands out as the most frequently occurring blood group within our study group. This observation aligns with a study conducted in proximity to the Asir region [23,24].

In our study, patients with blood type B+ had a 100% mortality rate, though their numbers were very small. Those with blood type O- had a mortality rate of 92.9%, while A+ and A- groups had rates of 62.5% and 58.5%, respectively. This analysis established significant statistical links, emphasizing the role of blood type in mortality risk. Remarkably, the only significant association with mortality was found in the Rh factor, where Rh-positive patients were nearly twice as likely to experience mortality compared to Rh-negative individuals. When examining this in the context of other studies conducted both at local and national levels, a definitive conclusion regarding the blood type most susceptible to COVID-19 remains elusive. Some studies have suggested that individuals with blood type A are at a higher risk of contracting the disease, while those with blood type O may be less prone to it [25-29]. However, conflicting findings have emerged from other research, with some studies failing to establish a significant link between blood type and COVID-19 susceptibility, disease duration, or severity [30-34].

Furthermore, research findings indicated that individuals with Rh-negative (Rh-) blood type exhibited a reduced risk of infection, intubation, and mortality, whereas patients with Rh-positive (Rh+) blood type demonstrated increased susceptibility to COVID-19. Nevertheless, a study conducted in Iran failed to establish a correlation between COVID-19 and Rh type [35-38]. Similarly, research conducted in Turkey did not identify any connection between Rh blood groups and their influence on the rates of hospitalization, ICU admission, mechanical ventilation support, or case fatality rates [39-40].

Finally, while our study provides valuable insights into the associations among blood group antigens, Rh types, and mortality in severely critical COVID-19 cases, we recognize certain limitations. The retrospective design, reliant on historical medical records, may result in incomplete data and biases. Furthermore, our investigation is restricted to a single center, limiting the broader generalizability of findings. Despite meticulous data cleansing, variations in data completeness and accuracy of medical records may persist.

## Conclusions

The variations in study outcomes could stem from differences in sample sizes, the diversity of ABO blood groups within various populations or regions, disparities in genetic backgrounds, and distinctions in viral strains. The divergence in blood group phenotypes among countries and genetic diversity may contribute to differences in the clinical manifestations of COVID-19.
